# Partially Divided Flexor Tendon Injuries: Should They Be Repaired or Not?

**DOI:** 10.1055/s-0036-1593356

**Published:** 2016-09-14

**Authors:** Shkelzen B. Duci, Hasan R. Ahmeti

**Affiliations:** 1 Clinic of Plastic Surgery, University Clinical Center of Kosovo, Prishtina, Kosovo; 2 Clinic of Pediatric Surgery, University Clinical Center of Kosovo, Prishtina, Kosovo

**Keywords:** flexor, lacerated, tendon

## Abstract

The correct management of partially divided flexor tendon injuries is still controversial. Opinions vary regarding whether partially divided flexor tendon injuries should be repaired. Flexor tendon injuries are common because the tendons lie close to the skin. The tendons are therefore exposed to injuries like lacerations and crush injuries, and occasionally they can rupture from where they are joined to the bone. Tendon injuries are the second most common hand injuries in orthopedic patients.

Flexor tendon injuries are common because the tendons lie close to the skin. Therefore, they are exposed to injuries like lacerations and crush injuries, and occasionally they can rupture at their bone insertions. 1 Tendon injuries are the second most common hand injuries in orthopedic patients. 2

The correct management of partially divided flexor tendon injuries is still controversial. Opinions regarding whether partially divided flexor tendon injuries should be repaired still vary across the literature. 3 Controversy also exists in the literature regarding the proper treatment of partial flexor tendon lacerations.

Wray et al created considerable controversy by recommending that partial flexor tendon lacerations be repaired. 4 5 Several authors have reported an increased risk of triggering, entrapment, or rupture associated with lacerations of the tendon and have advocated surgical repair of those injuries. 6 7 8 9 10

On the other hand, Bishop et al studied a canine model and described the relative effects of tenorrhaphy. They also described healing when the flexor tendon was not repaired. 6 Parameters evaluated after 35-day healing period included excursion, breaking strength, energy absorption, and stiffness. Data analysis in their study revealed statistically significant adverse effects on breaking strength, stiffness, and energy absorption when repaired by modified Kessler technique compared with the tendons that were not repaired, which resulted in tendon morphology closer to normal.

Similarly, McGeorge and Stilwell studied partially lacerated flexor tendon injuries and compared the results of repair with those of nonrepair, showing better results in tendons that were not repaired. 10 The authors concluded that partially lacerated tendons should not be repaired.

Also, the level of flexor tendon injury carries a prognostic implication because of the different anatomic constraints to the flexor tendons over their course from the muscle belly in the forearm to their insertion in the phalanges. Verdan developed a uniform nomenclature (zone I to zone V) that has now been accepted by most hand surgeons. 11

Many factors are unique to tendon injuries, especially in zone II, making it difficult to repair the tendons in this area. Most injuries in this zone that lacerate the tendon also disrupt the nutritional systems of the tendon that support recovery. Particularly, damage of the digital sheath leads to leakage of the synovial fluid contained within it. This loss of synovial fluid may starve the tendon repair process, because nutrients are normally provided to the tendon primarily via diffusion through this fluid. Furthermore, even if the loss of fluid is not great, damage in the sheath can impair tendon nutrition, because it allows the pressure of the fluid in the sheath to dissipate. This loss of pressure can deprive the tendon of nutrients, because the distribution of these nutrients is normally propelled by a process of imbibition, in which synovial fluid is forced into the interstices between tendon fascicles during flexion and extension of the digit. 12 13

Although there were more controversies among different authors regarding repair of flexor tendon digitorum superficialis and the flexor tendon digitorum profundus in zone II injury due to the creation of adhesions, most authors recommend that both tendons should be repaired, with the flexor digitorum superficialis tendon repaired first. 14

In brief, multiple investigators have concluded that partial lacerations involving ≤60% of the tendon's cross-sectional area should not be repaired. That recommendation is supported by both in vivo and ex vivo biomechanical studies that have demonstrated that nonrepaired partial lacerations bear significantly greater ultimate loads and exhibit greater stiffness than repaired tendons. 6 10 15

The recommendation of the majority of authors for injuries involving ≤60% of the tendon cross-sectional area is debridement of the tendon. Injuries involving >60% of the tendon should be repaired with traditional core-suture methods supplemented with a running epitendinous suture. 14

Future research on the treatment of the partially lacerated flexor tendons should focus on developments and understanding of soft tissue healing at the cellular, molecular, and genetic levels to enable surgeons to modulate the healing process to improve the strength of the repair site while at the same time reducing adhesion after injury and surgical repair.
